# Effects of Avocado Products on Cardiovascular Risk Factors in Adults: A GRADE‐Assessed Systematic Review and Meta‐Analysis

**DOI:** 10.1002/fsn3.70547

**Published:** 2025-07-02

**Authors:** Sahel Hamednia, Zahra Shouhani, Sara Tavakol, Nazanin Montazeri, Mohammadreza Amirkhan‐Dehkordi, Mohammad Amin Karimi, Mehraveh Bastamkhani, Haleh Chavoshian Tabrizy, Ramin Amirsasan, Javad Vakili, Mehdi Karimi, Omid Asbaghi

**Affiliations:** ^1^ Department of Exercise Physiology, Faculty of Physical Education and Sport Science University of Tabriz Tabriz Iran; ^2^ School of Medicine Ahvaz Jundishapur University of Medical Science Ahvaz Iran; ^3^ Department of Exercise Physiology Islamic Azad University, Central Tehran Branch Tehran Iran; ^4^ Department of Exercise Physiology University of Guilan Rasht Iran; ^5^ Department of Physical Education Sport Sciences Science and Research Islamic Azad University Tehran Iran; ^6^ School of Medicine, Shahid Beheshti University of Medical Sciences Tehran Iran; ^7^ Department of Nutrition, Science and Research Branch Islamic Azad University Tehran Iran; ^8^ Department for Sustainable Development and Ecological Transition (DISSTE) University of Eastern Piedmont Vercelli Italy; ^9^ Faculty of Physical Education and Sport Science University of Tabriz Tabriz Iran; ^10^ Faculty of Medicine, Bogomolets National Medical University (NMU) Kyiv Ukraine; ^11^ Cancer Research Center Shahid Beheshti University of Medical Sciences Tehran Iran; ^12^ Student Research Committee Shahid Beheshti University of Medical Sciences Tehran Iran

**Keywords:** avocado, nutrition, *Persea*: cardiovascular risk

## Abstract

Cardiovascular diseases are a major global health concern, and avocados, rich in monounsaturated fats and bioactive compounds, may help improve heart health by influencing lipid profiles and other risk factors. However, existing studies on avocado consumption and cardiovascular benefits show inconsistent results, and no comprehensive meta‐analysis has been conducted. This study aims to systematically review and analyze current research to provide a quantitative assessment of avocados' effects on cardiovascular risk factors in adults. From inception until May 2025, a comprehensive search was conducted on PubMed, Web of Science, and Scopus to find randomized controlled studies (RCTs) evaluating the effectiveness of avocado intake on cardiovascular risk factors. Following screening, data were extracted and analyzed by STATA. The pooled analysis of 10 RCTs showed that avocado intake had no significant change on triglycerides (TG) (WMD: 0.02 mg/dL; *p* = 0.97), total cholesterol (TC) (WMD: 1.28 mg/dL; *p* = 0.62), high‐density lipoprotein (HDL) (WMD: −0.27 mg/dL; *p* = 0.64), fasting blood glucose (FBG) (WMD: −0.05 mg/dL; *p* = 0.78), body mass index (BMI) (WMD: −0.07 kg/m^2^; *p* = 0.31) and C‐reactive protein (CRP) (WMD: 0.02 mg/dL; *p* = 0.06). Conversely, a significant reduction was observed in low‐density lipoprotein (LDL) (WMD: −3.75 mg/dL; *p* < 0.001; *I* ^2^ = 0%), systolic blood pressure (BP) (WMD: −1.15 mmHg; *p* = 0.03; *I* ^2^ = 56%), and an under border of insignificant change in diastolic BP (SWD: −0.03 mmHg; *p* = 0.066; *I* ^2^ = 61.9%). The findings from this meta‐analysis suggest that while avocado intake does not significantly impact triglycerides, total cholesterol, HDL, fasting blood glucose, BMI, or CRP, it is associated with a significant reduction in LDL and systolic blood pressure. These results indicate a potential cardioprotective effect of avocado consumption by lowering key risk factors for cardiovascular diseases. However, further well‐designed studies with larger sample sizes are needed to confirm these benefits and explore the long‐term effects of avocado intake on cardiovascular health.

## Graphical abstract

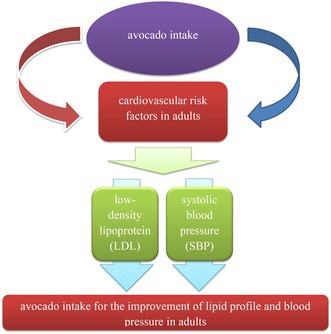

## 1 Introduction

Cardiovascular diseases (CVDs) create huge morbidity and mortality risks worldwide. They place a significant economic burden on the healthcare system (Vaduganathan et al. 2022). About 31% of deaths worldwide are ascribed to CVDs (Ruiz‐Moreno et al. 2020). Poor health behaviors and several illnesses, including dyslipidemia, hyperglycemia, hypertension, and inflammatory disorders, can raise the mortality rate from CVD linked to acute myocardial infarction and stroke (Hasanloei et al. 2020; Hashemi et al. 2020; Kopin and Lowenstein 2017; Noormohammadi et al. 2022). Since poor diet and lifestyle choices are a major contributing factor to CVDs, the main method for delaying the beginning of CVDs and their risk factors is to improve dietary habits and make them more widely available (Asbaghi, Fouladvand, et al. 2021; Asbaghi, Naeini, et al. 2021; Naseri et al. 2022). Given the advantages of nutrition in the development of CVD, alternative treatments like natural‐based products can help alleviate some CVD risk factors, such as hypertension, diabetes, and hypercholesterolemia (Mahmoud et al. 2019; Mas‐Capdevila et al. 2020) According to the current Dietary Guidelines for Americans, adults over the age of 18 should consume 20%–35% of their calories from fat. The recommendation encourages consuming PUFAs and/or MUFAs and fewer than 7% of calories from SFAs in order to lower blood cholesterol levels and the risk of cardiovascular disease (US Department of Agriculture and US Department of Health and Human Services 2010). Both guidelines recommend consuming fewer SFAs and substituting them with PUFAs and/or MUFAs (US Department of Agriculture and US Department of Health and Human Services 2010).

The avocado, scientifically named *Persea americana* , belongs to the Lauraceae family. It is classified as a large berry with a single large seed (Wegier et al. 2017). Avocados are rich in oleic acid, constituting about 52.11% of their total fatty acids. This high MUFA content is associated with cardiovascular health benefits, including improved lipid profiles and reduced LDL cholesterol levels. PUFA comprises about 13% of the total fat content; these include omega‐3 and omega‐6 fatty acids, essential for various bodily functions. Around 16% of the fatty acids in avocados are saturated fats (SFAs), primarily palmitic acid (approximately 41.56%) and stearic acid (about 0.1%) (Muralidhara et al. 2023). Monounsaturated fatty acid (MUFA), polyunsaturated fatty acids (PUFA), potassium, magnesium, dietary fiber, phytonutrients, and bioactive substances are all found in avocados, a fruit rich in nutrients that have been independently linked to cardiovascular health (Dreher and Davenport 2013; Liu et al. 2002).

Moreover, some studies have revealed that individuals consuming at least two servings of avocado per week exhibit a 16% lower risk of CVD and a 21% lower risk of coronary heart disease compared to non‐consumers. Specifically, those increasing their intake by half a serving per day had a pooled hazard ratio for CVD of 0.80, indicating a protective effect against heart disease (Pacheco et al. 2022). Besides, avocado consumption is important as a possible mechanism involved in protecting CVDs (Dreher and Davenport 2013; Liu et al. 2002). A thorough meta‐analysis of clinical trials (CTs) is necessary because the overall effect of avocado supplementation on cardiovascular disease is not entirely recognized. In order to ascertain the impact of avocado intake on cardiovascular risk factors, we set out to perform a systematic review and meta‐analysis.

## 2 Methods

### 2.1 Study Design and Protocol

The Preferred Reporting Items of Systematic Reviews and Meta‐Analysis (PRISMA) statement standards were followed in the conduct of this study (Page et al. 2021).

### 2.2 Search Strategy

A comprehensive search was conducted in PubMed, Scopus, ISI Web of Science, and Cochrane Library, and a manual search of the bibliographies of the Persian and English articles retrieved until May 2025. The search for text words and controlled vocabulary phrases was conducted using medical subject headings (MeSH). The merger of MeSH and non‐MESH terms was as follows: (“Avocado” OR “Persea” OR “ *Persea americana* ” OR “Alligator pear”) AND (“randomized” OR “random” OR “randomly” OR “placebo” OR “randomized controlled trial” OR “randomized clinical trial” OR “RCT” OR “blinded” OR “double blind” OR “double blinded” OR “Cross‐Over” OR “parallel”) with no language or date restrictions (Table S1). To prevent missing any related studies, we also hand‐searched all reference lists of eligible studies, related reviews, and meta‐analyses.

### 2.3 Inclusion Criteria

Studies with the following conditions were included in this meta‐analysis: (a) RCTs and (b) performed on adults (≥ 18 year). All studies that examined the effects of avocado supplementation on body weight, BMI, SBP, DBP, blood levels of FBG, HbA1c, TG, fasting insulin, LDL, CRP, TC, and HDL in the avocado group relative to the placebo group were included in the current systematic review and meta‐analysis.

### 2.4 Excluded Criteria

At most one of the following traits applied to excluded studies: Research that (a) lacked randomized controlled trials (RCTs), (b) looked at avocado's effects in combination with other interventions, (c) provided insufficient details for the key outcomes, (d) had follow‐ups less than a week, (e) animal studies, (f) case reports, (g) conference abstracts, and (h) articles with unclear data descriptions.

### 2.5 Quality Assessment

The 5‐item Cochrane risk of bias 2 (RoB 2) method was used by two reviewers (S.H) to assess the trial's quality (Sterne et al. 2019). ROB2 items include: (1) randomization process; (2) deviations from the intended interventions; (3) missing outcome data; (4) measurement of the outcome; and (5) selection of the reported result.

### 2.6 Data Extraction

Two authors independently (S.H. and Z.S.) assessed the quality of each included study using the Cochrane risk of bias tool in Review Manager. The study's design, duration, location, sample size, first author, year of publication, and dosage of the avocado supplement are among its specifics. Initial and final measures of certain outcomes mean differences in outcome changes between the study's initial and final measurements, as well as participant demographics, such as gender, average age, BMI, and comorbidities. The main outcomes were body weight, BMI, SBP, DBP, and blood levels of TG, fasting insulin, HbA1c, LDL, CRP, FBG, TC, and HDL. Additionally, two scholars resolved disagreements and discrepancies through communication and consultation with a third author (O.A.).

### 2.7 Statistical Analysis

This meta‐analysis was conducted using Stata software (version 17). To assess the effect of avocado, weighted mean differences (WMDs) and 95% CIs were calculated to measure the absolute changes in outcomes between the avocado and placebo groups from the trial baseline to endpoints. The results were quantified using the mean ± standard deviation (SD) measurement, and the magnitude of the effect was calculated by computing the mean difference. The formula square root ([SD2 baseline + SD2 final] − [2 × *R* × SDbaseline × SDfinal]) was used to determine the SD change from the trial's baseline to its conclusion (Borenstein et al. 2021). Furthermore, a random‐effects model was used to compute the pooled WMDs (DerSimonian and Laird 1986). The between‐study heterogeneity was assessed using the *I* ^2^ statistic (Borenstein et al. 2010), which showed moderate, low, and high degrees of heterogeneity.

The analysis considered baseline values of HDL, TC, FBG, TG, LDL, SBP, BMI, WC, and CRP serum levels; avocado dosage (≥ 300 mg/day vs. < 300 mg/day); baseline BMI (obese [≥ 30 kg/m^2^] vs. normal [18.5–24.9 kg/m^2^]; trial duration [< 6 weeks vs. ≥ 6 weeks]; and health status [CVD vs. none CVD]). A leave‐one‐out sensitivity analysis was used to assess the influence of particular research on the findings. A *p*‐value of less than 0.05 was established as the threshold for statistical significance. Additionally, funnel plots and the Egger's (Egger et al. 1997) and Begg's (Begg and Berlin 1988) tests were used to look for any publication bias. The fractional polynomial model was also used to analyze the potential non‐linear effects of the trial's duration (weeks) and avocado dosage (mg/day). To assess the link between dose and response and potential linear effects (Mitchell 2012).

## 3 Result

### 3.1 Study Selection

**FIGURE 1:**
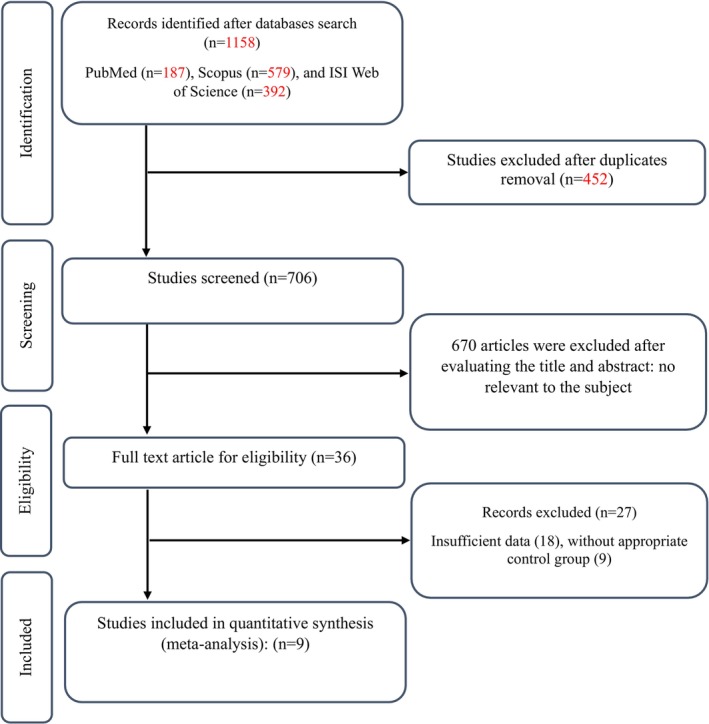
Flow chart of study selection for inclusion trials in the systematic review.

The study selection process began with the identification of 1158 records from databases, including PubMed (*n* = 187), Scopus (*n* = 579), and ISI Web of Science (*n* = 392). After removing 452 duplicate records, 706 records were initially screened based on titles and abstracts. A total of 670 records were excluded due to being review studies, non‐human studies, or having irrelevant titles and abstracts. Following this, 36 full‐text articles were assessed for eligibility, and 17 studies that did not meet the inclusion criteria were excluded. Ultimately, nine (Hernández Salazar et al. 2022; Lichtenstein et al. 2022; Martínez‐Abundis et al. 2013; Matthan et al. 2024; Pieterse et al. 2005; Scott et al. 2017; Souza Fernandes Azevedo et al. 2023; Zhang et al. 2022; Zhao et al. 2023) RCTs were included in the systematic review and meta‐analysis (Figure 1).

### 3.2 Study Characteristics

The characteristics of the included RCTs are summarized in Table 1. This meta‐analysis incorporates data from 10 RCTs with a total of 2354 participants (1181 cases and 1173 controls), published between 2005 and 2024. All of the studies followed a randomized parallel design (Hernández Salazar et al. 2022; Lichtenstein et al. 2022; Martínez‐Abundis et al. 2013; Matthan et al. 2024; Pieterse et al. 2005; Scott et al. 2017; Souza Fernandes Azevedo et al. 2023; Zhang et al. 2022; Zhao et al. 2023). Sample sizes ranged from 13 to 1008 participants, with trial durations spanning 4–24 weeks. Participants' average ages ranged from 32 to 63 years. The trials were conducted in the USA (Lichtenstein et al. 2022; Matthan et al. 2024; Scott et al. 2017; Zhang et al. 2022), Australia (Zhao et al. 2023), Mexico (Martínez‐Abundis et al. 2013), Brazil (Souza Fernandes Azevedo et al. 2023), and South Africa (Pieterse et al. 2005), and metabolic syndrome (Souza Fernandes Azevedo et al. 2023), overweight and obese (Lichtenstein et al. 2022; Martínez‐Abundis et al. 2013; Matthan et al. 2024; Pieterse et al. 2005; Zhao et al. 2023), and healthy individuals (Hernández Salazar et al. 2022). Nine studies involving both sexes (Hernández Salazar et al. 2022; Lichtenstein et al. 2022; Matthan et al. 2024; Pieterse et al. 2005; Scott et al. 2017; Souza Fernandes Azevedo et al. 2023; Zhang et al. 2022; Zhao et al. 2023), and one involving women was conducted (Martínez‐Abundis et al. 2013). The avocado supplements consumed daily ranged from 25 to 300 g.

**TABLE 1:** Basic characteristics of the included studies.

Studies	Country	Study design	Participant	Sample size and gender	Sample size		Trial duration (weeks)	Means age		Means BMI		Intervention	
					IG	CG		IG	CG	IG	CG	Avocado dose (mg/day)	Control group
Pieterse et al. (2005)	South Africa	R, PC, SB (parallel)	Obesity	61 (F: 13, M: 47)	31	30	6	40.8 ± 8.94	40.8 ± 8.94	31.9 ± 3.9	31.9 ± 3.9	2000	Placebo
Martínez‐Abundis et al. (2013)	Mexico	R, PC, DB (parallel)	Obesity	14 (F: 14)	7	7	12	35.4 ± 4.3	35.4 ± 3.8	35.6 ± 2.8	34.5 ± 3.5	300	Placebo
Scott et al. (2017)	USA	R, PC (parallel)	Healthy	40 (F: 25, M: 15)	20	20	24	63.3 ± 11.1	62.5 ± 9.2	24.1 ± 3.1	24.2 ± 2.4	135,000	Placebo
Hernández Salazar et al. (2022)	Mexico	R, PC (parallel)	Healthy	13 (F: 8, M: 5)	6	7	4	32 ± 11.2	32 ± 5.4	22.3 ± 2.3	21.2 ± 2.2	25,000	Placebo
Lichtenstein et al. (2022)	USA	R, PC (parallel)	Obesity	1008 (F: 730, M: 278)	505	503	24	50.1 ± 14.3	50.4 ± 13.8	32.9 ± 5.3	33.2 ± 5.6	NR	Placebo
Zhang et al. (2022)	USA	R, PC (parallel)	Overweight and obese	93 (F: 54, M: 39)	49	44	12	40.6 ± 11.8	42.7 ± 12.5	32.3 ± 3.90	32.8 ± 3.88	NR	Placebo
Zhao et al. (2023)	Australia	R PC, DB (parallel)	Obesity	60 (F: 47, M: 13)	29	31	12	51 ± 12	46 ± 13	33.8 ± 2.7	34.2 ± 2.5	10,000	Placebo
Souza Fernandes Azevedo et al. (2023)	Brasil	R, PC, DB (paralell)	Metabolic syndrome	31 (F: 27, M: 4)	14	17	12	30–60	30–60	39.83 ± 6.98	39.57 ± 6.89	NR	Placebo
Matthan et al. (2024)	USA	R, PC (parallel)	Obesity	994 (F: 721, M: 273)	500	494	24	49.9 ± 14.3	50.5 ± 13.7	32.9 ± 5.3	33.2 ± 5.6	NR	Placebo

Abbreviations: CG, control group; CO, cross over; DB, double‐blinded; F, female; IG, intervention group; M, male; NR, not reported; PC, placebo‐controlled; RA, randomized; SB, single‐blinded.

### 3.3 Meta‐Analysis

#### 3.3.1 Effect of Avocado Supplementation on TG

**FIGURE 2:**
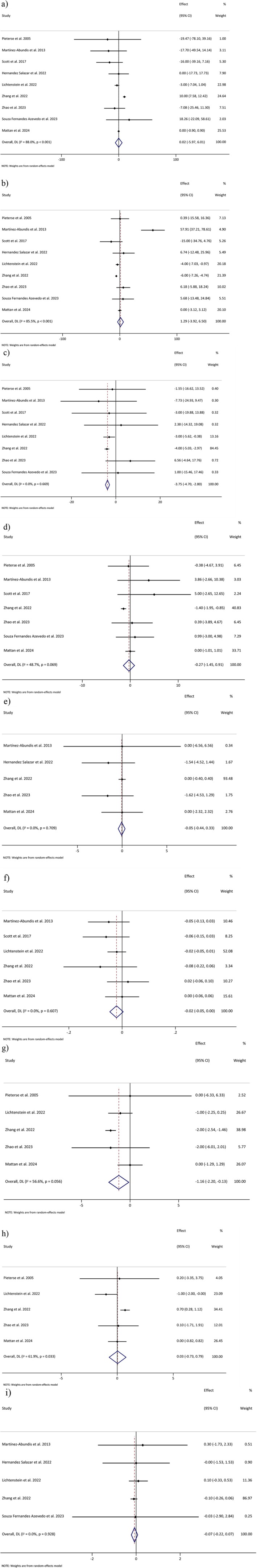
Forest plots for the effect of avocado supplementation on (a) TG (mg/dL), (b) TC (mg/dL), (c) LDL (mg/dL), (d) HDL (mg/dL), (e) FBG (mg/dL), (f) CRP (mg/dL), (g) SBP (mmHg), (h) DBP (mmHg), and (i) BMI (kg/m^2^). Diamonds represent pooled estimates from random‐effects analysis. The effect column comprises weighted mean differences (WMDs) and 95% CIs.

A meta‐analysis of nine studies involving 2314 participants found that avocado intake had no significant effect on TG levels (WMD: 0.02 mg/dL, 95% CI: −5.97 to 6.01, *p* = 0.64) (Figure 2). High between‐study heterogeneity was observed (*I* ^2^ = 88%, *p* < 0.001). Subgroup analysis further confirmed that avocado supplementation did not reduce TG levels (Table 2; Figure 2a).

**TABLE 2:** Subgroup analysis of the impacts of avocado supplementation on cardiovascular risk factors.

	Number of study	WMD (95% CI)	*p*	Heterogeneity		
				*p* heterogeneity	*I* ^2^ (%)	*p* between sub‐groups
Subgroup analyses of avocado supplementation on TG						
Overall effect	9	0.02 (−5.93, 6.09)	**0.97**	< 0.001	88.0	
Baseline TG (mg/dL)						
< 150	7	−0.12 (−6.27, 6.02)	0.96	< 0.001	90.8	0.75
≥ 150	2	5.63 (−29.25, 40.52)	0.75	< 0.001	7.4	
Trial duration (weeks)						
≤ 6	2	−1.63 (−18.59, 15.33)	**0.85**	0.53	0.0	0.83
> 6	7	0.25 (−6.15, 6.65)	0.93	< 0.001	90.9	
Intervention dose (mg/day)						
< 300	1	−17.70 (−49.54, 14.14)	0.27	—	—	0.52
≥ 300	4	−6.78 (−18.07, 4.49)	0.23	0.71	0.0	
Health status						
CVD	1	−0.31 (−6.40, 5.78)	0.37	—	—	0.37
Non‐CVD	8	18.26 (−22.09, 58.61)	0.92	< 0.001	89.4	
Sex						
Both sexes	8	0. 65 (−5.44, 6.76)	0.83	< 0.001	89.3	0.26
Female	1	−17.70 (−49.54, 14.14)	0.27	—	—	
Baselin BMI (kg/m^2^)						
Normal (18.5–24.9)	2	−6.19 (−21.46, 9.08)	0.42	0.28	13.5	0.39
Obese (> 30)	7	1.06 (−5.45, 7.57)	0.75	< 0.001	90.7	
Subgroup analyses of avocado supplementation on TC						
Overall effect	9	1.28 (−3.92, 6.49)	0.62	< 0.001	85.5	
Baseline TC (mg/dL)						
< 200	7	2. 39 (−3.26, 8.06)	0.40	< 0.001	88.7	0.29
≥ 200	2	−6.16 (−21.07, 8.75)	0.41	0.23	29.0	
Trial duration (weeks)						
≤ 6	2	1.02 (−4.63, 6.68)	0.63	0.61	0.0	0.77
> 6	7	2.98 (−9.30, 15.26)	0.72	< 0.001	88.7	
Intervention dose (mg/day)						
< 300	1	57.91 (37.21, 78.60)	**< 0.001**.	—	—	0.00
≥ 300	4	0.93 (−7.92, 9.79)	0.83	0.32	13.5	
Health status						
CVD	1	1. 23 (−3.98, 6.45)	0.71	—	—	0.64
Non‐CVD	8	0. 99 (−4.40, 6.38)	0.56	< 0.001	86.9	
Sex						
Both sexes	8	−2.61 (−5.94, 0.71)	0.14	0.006	64.1	0.00
Female	1	57.91 (37.21, 78.60)	**< 0.001**	—	—	
Baselin BMI (kg/m^2^)						
Normal (18.5–24.9)	2	−4.00 (−25.30, 17.30)	0.71	0.12	58.2	0.60
Obese (> 30)	7	1. 89 (−3.66, 7.45)	0.50	< 0.001	88.5	
Subgroup analyses of avocado supplementation on LDL						
Overall effect	8	−3.75 (−4.70, −2.80)	**< 0.001**	0.669	0.0	
Baseline LDL (mg/dL)						
< 100	1	2. 38 (−14.31, 19.07)	0.78	—	—	0.47
≥ 100	7	−3.78 (−4.73, −2.83)	**< 0.001**	0.68	0.0	
Trial duration (weeks)						
≤ 6	2	0. 21 (−10.97, 11.40)	0.97	0.73	0.0	0.48
> 6	6	−3.79 (−4.74, −2.84)	**< 0.001**	0.57	0.0	
Intervention dose (mg/day)						
< 300	1	−7.73 (−24.93, 9.47)	0.37	—	—	0.31
≥ 300	4	1. 95 (−5.44, 9.34)	0.60	0.77	0.0	
Health status						
CVD	1	−3.76 (−4.71, −2.81)	0.90	—	—	0.57
Non‐CVD	7	−3. 78 (−4.73, −2.82)	**< 0.001**	0.66	0.0	
Sex						
Both sexes	7	−3.75 (−4.70, −2.80)	**< 0.001**	0.64	0.0	0.65
Female	1	−7.73 (−24.93, 9.47)	0.37	—	—	
Baselin BMI (kg/m^2^)						
Normal (18.5–24.9)	2	−0. 28 (−12.15, 11.59)	0.96	0.65	0.0	0.56
Obese (> 30)	6	−3.78 (−4.74, −2.83)	**< 0.001**	0.56	0.0	
Subgroup analyses of avocado supplementation on HDL						
Overall effect	7	−0.27 (−1.45, 0.90)	0.64	0.07	48.7	
Baseline HDL (mg/dL)						
< 50	4	−0.64 (−2.27, 0.98)	0.43	0.26	24.5	0.44
≥ 50	3	−0.27 (−1.45, 0.90)	0.84	0.44	0.0	
Trial duration (weeks)						
≤ 6	1	−0.27 (−1.45, 0.90)	0.86	—	—	0.93
> 6	6	−0. 19 (−1.50, 1.11)	0.76	0.04	56.8	
Intervention dose (mg/day)						
< 300	1	3. 86 (−2.66, 10.38)	0.24	—	—	0.38
≥ 300	3	0. 70 (−2.21, 3.61)	0.63	0.47	0.0	
Health status						
CVD	1	0. 99 (−2.99, 4.97)	0.62	—	—	0.52
Non‐CVD	6	−0.35 (−1.61, 0.89)	0.57	0.05	53.3	
Sex						
Both sexes	6	−0.45 (−1.57, 0.66)	0.42	0.09	47.5	0.20
Female	1	3. 86 (−2.66, 10.38)	0.24	—	—	
Baselin BMI (kg/m^2^)						
Normal (18.5–24.9)	1	−0.27 (−1.45, 0.90)	0.20	—	—	0.16
Obese (> 30)	6	−0.46 (−1.55, 0.63)	0.41	0.09	46.2	
Subgroup analyses of avocado supplementation on FBG						
Overall effect	5	−0.05 (−0.43, 0.33)	0.78	0.70	0.0	
Baseline FBG (mg/dL)						
< 100	3	−1.43 (−3.42, 0.54)	0.15	0.90	0.0	0.16
≥ 100	2	0. 00 (−0.39, 0.39)	1.00	1.00	—	
Trial duration (weeks)						
≤ 6	1	−1.54 (−4.52, 1.44)	0.31	—	—	0.32
> 6	4	−0. 02 (−0.41, 0.35)	0.88	0.76	0.0	
Baselin BMI (kg/m^2^)						
Normal (18.5–24.9)	1	−1.54 (−4.52, 1.44)	0.31	—	—	0.32
Obese (> 30)	4	−0.02 (−0.41, 0.36)	0.88	0.76	0.0	
Subgroup analyses of avocado supplementation on SBP						
Overall effect	5	−1.15 (−2.19, −0.11)	**0.03**	0.05	56.6	
Baseline SBP (mmHg)						
< 120	1	0. 00 (−6.32, 6.32)	1.00	—	—	0.72
≥ 120	4	−1.15 (−2.19, −0.11)	**0.03**	0.03	66.5	
Trial duration (weeks)						
≤ 6	1	−1. 15 (−2.19, −0.11)	1.00	—	—	0.72
> 6	4	−1. 17 (−2.28, −0.06)	**0.03**	0.03	66.5	
Subgroup analysis of avocado supplementation on DBP						
Overall effect	6	−0.03 (−0.07, 0.00)	0.06	0.003	61.9	
Baseline DBP (mmHg)						
< 80	2	−0.01 (−0.09, 0.06)	0.68	0.18	42.2	0.61
≥ 80	4	−0.04 (−0.08, 0.00)	0.06	0.00	78.0	
Intervention dose (mg/day)						
< 300	1	−0. 05 (−0.12, 0.02)	0.20	—	—	0.57
≥ 300	2	−0.03 (−0.08, 0.02)	0.65	0.18	42.2	
Sex						
Both sexes	5	−0.03 (−0.07, 0.01)	0.13	0.00	76.9	0.71
Female	1	−0.05 (−0.12, 0.02)	0.20	—	—	
Baselin BMI (kg/m^2^)						
Normal (18.5–24.9)	1	−0.03 (−0.07, 0.00)	0.18	—	—	0.58
Obese (> 30)	5	−0.03 (−0.07, 0.01)	0.14	0.00	77.0	
Subgroup analysis of avocado supplementation on BMI						
Overall effect	5	−0.07 (−0.21, 0.07)	0.31	0.92	0.0	
Baseline BMI (kg/m^2^)						
< 30	1	−0.00 (−1.52, 1.52)	1.00	—	—	0.92
≥ 30	4	−0.07 (−0.22, 0.07)	0.31	0.83	0.0	
Trial duration (weeks)						
≤ 6	1	−0.07 (−0.21, 0.07)	1.00	—	—	0.92
> 6	4	−0.07 (−0.22, 0.07)	0.31	0.83	0.0	
Intervention dose (mg/day)						
< 300	1	0. 30 (−1.73, 2.33)	0.77		—	0.93
≥ 300	1	0. 00 (−1.52, 1.52)	1.00	—	—	
Health status						
CVD	1	−0. 07 (−0.21, 0.07)	0.98	—	—	0.97
Non‐CVD	4	−0. 07 (−0.21, 0.07)	0.31	0.83	0.0	
Sex						
Both sexes	4	−0.07 (−0.22, 0.06)	0.30	0.86	0.0	0.71
Female	1	0. 30 (−1.73, 2.33)	0.77	—	—	
Baselin BMI (kg/m^2^)						
Normal (18.5–24.9)	1	−0.07 (−0.21, 0.07)	1.00	—	—	0.92
Obese (> 30)	4	−0.07 (−0.22, 0.07)	0.31	0.83	0.0	
Subgroup analysis of avocado supplementation on CRP						
Overall effect	6	0.02 (−0.04, 0.00)	0.10	0.60	0.0	
Baseline CRP						
> 0.3	4	−0.02 (−0.05, 0.00)	0.11	0.63	0.0	0.89
< 0.3	2	−0.01 (−0.09, 0.06)	0.68	0.17	46.2	
Intervention dose (mg/day)						
< 300	2	−0.01 (−0.09, 0.06)	0.68	0.17	46.2	0.55
≥ 300	1	−0.05 (−0.12, 0.02)	0.20	—	—	
Sex						
Both sexes	5	−0.01 (−0.04, 0.00)	0.67	0.55	0.0	0.44
Female	1	−0.05 (−0.12, 0.02)	0.20	—	—	
Baselin BMI (kg/m^2^)						
Normal (18.5–24.9)	1	−0.06 (−0.14, 0.02)	0.18	—	—	0.36
Obese (> 30)	5	−0.01 (−0.04, 0.00)	0.19	0.59	0.0	

*Note*: Bold value indicates statistical significant (*p* < 0.05).

Abbreviations: CI, confidence interval; WMD, weighted mean differences.

#### 3.3.2 Effect of Avocado Supplementation on TC

The pooled effect sizes from nine RCTs involving 2314 people (1161 cases and 1153 controls) showed that avocado supplementation did not significantly change TC (WMD: 1.29 mg/dL; 95% CI: −3.92 to 6.50; *p* = 0.62), with a substantial heterogeneity (*I* ^2^ = 85.5%) (Figure 2b). The subgroup analysis was carried out according to gender, serum baseline TC, baseline BMI, and duration of the intervention to identify the source of heterogeneity. As a result, subgroup analysis revealed that avocado supplementation did not reduce TC levels (Table 2).

#### 3.3.3 Effect of Avocado Supplementation on LDL

Nine studies with 2314 participants were analyzed overall. The results showed that those who took avocado supplements had lower LDL levels than the control group (WMD: −3.75 mg/dL, 95% CI: −4.70 to −2.80; *p* < 0.001) (Figure 2c), with between‐study heterogeneity (*I* ^2^ = 0%, *p* = 0.669). The subgroup analysis based on the baseline LDL showed that the decrease in LDL following avocado supplementation was significant at LDL > 100 (WMD: −3.78 mg/dL; 95% CI: −4.73, −2.83; *p* < 0.001). Additionally, other subgroup analyses revealed that avocado significantly decreased LDL in non‐CVD patients (WMD: −3.78 mg/dL, 95% CI: −4.73, −2.82; *p* < 0.001) and in long‐duration of intervention (> 6 weeks) studies in both sexes (WMD: −3.75 mg/dL, 95% CI: −4.70, −2.80; *p* < 0.001) and individuals with BMI > 30 (WMD: −3.78 mg/dL, 95% CI: −4.74, −2.83; *p* < 0.001).

#### 3.3.4 Effect of Avocado Supplementation on HDL

The analysis includes a total of 2314 participants from nine different trials. Avocado supplementation had a lowering but non‐statistically significant effect on HDL (WMD: −0.27 mg/dL; 95% CI: −1.45, 0.91; *p* = 0.064), according to the meta‐analysis (Figure 2d). Between‐study heterogeneity (*I* ^2^ = 48.7%, *p* = 0.069) was found. In all subgroups, our subgroup analysis revealed no significant between‐study heterogeneity (Table 2).

#### 3.3.5 Effect of Avocado Supplementation on FBG

**FIGURE 3:**
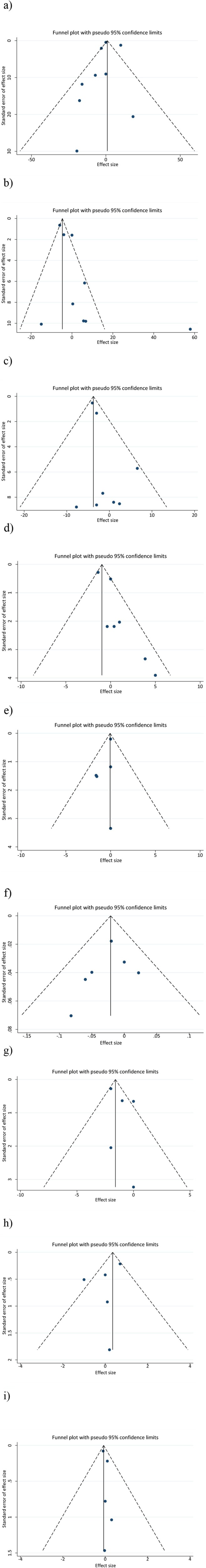
Funnel plots for the effect of avocado consumption on (a) TG (mg/dL), (b) TC (mg/dL), (c) LDL (mg/dL), (d) HDL (mg/dL), (e) FBG (mg/dL), (f) CRP (mg/dL), (g) SBP (mmHg), (h) DBP (mmHg), and (i) BMI (kg/m^2^).

The meta‐analysis revealed a lowering but non‐statistically significant effect of avocado supplementation on FBG (WMD: −0.05 mg/dL; 95% CI: −0.44, 0.33; *p* = 0.79). The analysis comprised six trials with a total of 1174 participants. Between heterogeneity (*I* ^2^ = 0.0%, *p* = 0.709) was found (Table 2; Figure 3e).

#### 3.3.6 Effect of Avocado Supplementation on CRP

The overall analysis of six studies enrolling 1174 participants showed no significant changes in CRP among individuals assigned to avocado supplementation compared with controls (WMD: −0.02 mg/dL; 95% CI: −0.05, 0.00; *p* = 0.06) (Figure 2f), with between‐study heterogeneity (*I* ^2^ = 0.0%, *p* = 0.607). Our subgroup analysis showed no significant between‐study heterogeneity in all subgroups (Table 2).

#### 3.3.7 Effect of Avocado Supplementation on SBP

Five clinical trials totaling 2216 people were included in our meta‐analysis. With considerable between‐study heterogeneity (*I* ^2^ = 56.6%, *p* = 0.056), we combined these effect sizes to find that avocado supplementation had a significant effect on SBP (WMD: −1.16 mmHg; 95% CI: −2.20, −0.13; *p* = 0.03) (Figure 2g). Subgroup analysis showed that the between‐study heterogeneity was explained by the intervention duration and baseline SBP. We observed a significant effect of avocado supplementation on SBP in studies that included SBP > 120 mmHg (WMD: −1.15 mmHg; 95% CI: −2.19, −0.11; *p* = 0.03) and those with a duration of intervention > 6 weeks (WMD: −1.17 mmHg; 95% CI: −2.28, −0.06; *p* = 0.03) (Table 2).

#### 3.3.8 Effect of Avocado Supplementation on DBP

Five clinical trials with a total of 2216 people were included in our meta‐analysis. When combining these effect sizes, we found that avocado supplementation had increased DBP but was not statistically significant (WMD: 0.03 mmHg; 95% CI: −0.73, 0.79; *p* = 0.938), with significant between‐study heterogeneity (*I* ^2^ = 61.9%; *p* = 0.033) (Figure 2h). Subgroup analysis showed that the between‐study heterogeneity was explained by sex, health status, baseline BMI, intervention dose, and duration of the intervention (Table 2).

#### 3.3.9 Effect of Avocado Supplementation on BMI

A total of 1151 participants (581 cases and 578 controls) participated in five trials examining how avocado supplementation affected BMI. Avocado supplementation did not significantly affect BMI, according to the overall effect sizes (WMD: −0.07 mmHg; 95% CIs: −0.22 to 0.07; *p* = 0.31) (Figure 2i). The degree of heterogeneity (*I* ^2^ = 0%, *p* = 0.928) was found. Gender, study participants' health state, baseline BMI, length of intervention, and dosage were determined to account for 0.0% of the heterogeneity in the subgroup analysis. When considering the overall impacts, subgroup analysis revealed that the results remained significant (Table 2).

### 3.4 Sensitivity Analysis

To ascertain each study's impact on the overall effect size, we omitted each trial from the analysis step by step. After deleting the study of Zhang et al. (2022) the overall effect of avocado on HOMA‐IR significantly changed (WMD: −109.76, 95% CI: −106.76, 78.24). For SBP, after removing the study by Zhao et al. (2023), Lichtenstein et al. (2022) and Zhang et al. (2022), the overall effect changed significantly. Based on the results of the sensitivity analysis, data on DBP was sensitive to studies by Lichtenstein et al. (2022) (WMD: 0.53, 95% CI: 0.16, 0.90), and the overall results changed to significant. The overall effect of avocado on CRP also changed to a significant value after excluding studies by Zhao et al. (2023) (WMD: 0.04, 95% CI: −0.08, −0.005) and Matthan et al. (2024) (WMD: −0.04, 95% CIs: −0.08, −0.002).

### 3.5 Publication Bias and Risk of Bias Assessment

Despite some publication bias asymmetry seen by visual inspection of funnel plots the number of included studies was less than 10. Therefore, we did not perform publication bias tests based on the Egger's and Begg's tests (Figure 3). The ROB 2 across the studies included in this meta‐analysis is detailed in Table 3.

**TABLE 3:** Risk of bias assessment 2.

Study	D1	D2	D3	D4	D5	Overall
Pieterse et al. (2005)	L	H	H	H	L	Moderate‐risk
Martínez‐Abundis et al. (2013)	L	L	L	L	L	Low‐risk
Scott et al. (2017)	L	H	H	L	L	Moderate‐risk
Hernández Salazar et al. (2022)	L	H	H	H	L	Low‐risk
Lichtenstein et al. (2022)	L	H	H	H	L	Low‐risk
Zhang et al. (2022)	L	H	H	H	L	Low‐risk
Zhao et al. (2023)	L	L	L	H	L	Moderate‐risk
Souza Fernandes Azevedo et al. (2023)	L	L	L	H	L	Low‐risk
Matthan et al. (2024)	L	H	H	H	L	Low‐risk

*Note:* Risk of bias domains: D1, randomization process; D2, deviations from the intended interventions; D3, missing outcome data; D4, measurement of the outcome; D5, selection of the reported result.

### 3.6 GRADE Assessment

The GRADE assessment of avocado's effects on CVD risk factors in adults, as presented in Table 4, indicates varying levels of evidence quality across different outcomes. For TG and TC, the evidence quality is rated as low due to very serious inconsistency and serious imprecision, despite no serious limitations in risk of bias or indirectness. High‐quality evidence was found for LDL, with no limitations in any domains, whereas high evidence was also observed for FBG, SBP, BMI, and CRP, though these outcomes were impacted by serious imprecision. DBP had moderate‐quality evidence due to serious limitations in both inconsistency and imprecision. High limitations due to inconsistency and imprecision reduced the quality for HDL, which also had significant publication bias. Overall, the quality of evidence ranged from very high to low, with notable concerns around heterogeneity, imprecision, and publication bias affecting some outcomes.

**TABLE 4:** Grade profile of avocado for CVD risk factors in adults.

Outcomes	Risk of bias	Inconsistency	Indirectness	Imprecision	Publication bias	Quality of evidence
TG	No serious limitation	Very serious limitation^a^	No serious limitation	Serious limitation^c^	No serious limitation	⊕◯◯◯ Low
TC	No serious limitation	Very serious limitation^a^	No serious limitation	Serious limitation^c^	No serious limitation	⊕◯◯◯ Low
LDL	No serious limitation	No serious limitation	No serious limitation	No serious limitation	No serious limitation	⊕⊕⊕⊕ Very High
HDL	No serious limitation	Serious limitation^b^	No serious limitation	Serious limitation^c^	Serious limitation^d^	⊕◯◯◯ Low
FBG	No serious limitation	No serious limitation	No serious limitation	Serious limitation^c^	No serious limitation	⊕⊕⊕◯ High
SBP	No serious limitation	Serious limitation^b^	No serious limitation	No serious limitation	No serious limitation	⊕⊕⊕◯ High
DBP	No serious limitation	Serious limitation^b^	No serious limitation	Serious limitation^c^	No serious limitation	⊕⊕◯◯ Moderate
BMI	No serious limitation	No serious limitation	No serious limitation	Serious limitation^c^	No serious limitation	⊕⊕⊕◯ High
CRP	No serious limitation	No serious limitation	No serious limitation	Serious limitation^c^	No serious limitation	⊕⊕⊕◯ High

^a^ There is high heterogeneity (*I* ^2^ > 75%).

^b^ There is moderate heterogeneity (*I* ^2^ > 40%).

^c^ There is no evidence of significant effects of avocado intake.

^d^ There is a significant publication bias based on Egger's test.

## 4 Discussion

This study aimed to assess the effects of avocado consumption on lipid profiles, blood glucose, inflammatory markers, and blood pressure to determine its impact on cardiovascular disease risk factors. Our pooled analysis of 10 RCTs suggests that avocado intake does not significantly affect triglycerides, total cholesterol, HDL, fasting blood glucose, BMI, or C‐reactive protein. However, a notable reduction in LDL cholesterol and systolic blood pressure was observed, with a borderline insignificant decrease in diastolic blood pressure. These findings suggest that while avocado consumption may provide some cardiovascular benefits, particularly in lowering LDL cholesterol and systolic BP, it does not appear to have a substantial effect on other metabolic and inflammatory markers.

Our findings are supported by recent research highlighting the importance of avocados in reducing cardiovascular risk factors. In a randomized study, for example, Wang et al. (2015) discovered that eating one avocado daily for 6 weeks raised plasma lutein concentrations and decreased circulating LDL levels. A high‐carb, low‐fat diet, a moderate‐fat diet with corresponding macronutrients and fatty acids, and a normal Western diet contrasted with this impact. Beyond their fatty acids, avocado's bioactive components were thought to be responsible for the advantages.

On the avocado diet, oxLDL may have decreased due to the decrease in small LDL particles. Moreover, even on a low‐fat, high‐carb diet that raises small, dense LDL (sdLDL) particles, increased consumption of fruits, vegetables, and whole grains may offer protection against the oxidation of atherogenic lipoproteins (Wang et al. 2020).

According to a thorough study on American men and women by Pacheco et al. (2022), eating avocados regularly can dramatically lower the incidence of coronary heart disease and CVDs. However, there was no significant link between eating avocados and either ischemia or total stroke. According to the study, avocados can reduce the incidence of cardiovascular disease by replacing unhealthy foods, including butter, margarine, processed meats, cheese, and eggs. This research offers more proof that including plant‐based unsaturated fats in our diet can enhance its quality and significantly lower the risk of CVD in the general population.

Beyond its fatty acid composition, Wang et al. (2020) discovered that eating one Hass avocado daily can help lower LDL and other newly identified risk factors for CVD. This study also demonstrates that when compared to a high‐monounsaturated‐fat diet with comparable macronutrient and fatty acid profiles, a moderate‐fat diet low in saturated fat and high in monounsaturated fat from avocados can result in larger reductions in a variety of cholesterol indicators. Therefore, in comparison to a low‐saturated‐fat diet with equivalent amounts of monounsaturated fats, including a food source high in monounsaturated fats and bioactive substances can offer further cardiovascular advantages.

Our analysis revealed that supplementing with avocados did not significantly alter glucose levels in terms of FBG levels after 6 months of consuming one avocado per day. Lichtenstein et al. (2022) found no discernible differences in the FBG between the HD and avocado diets. Furthermore, Wang et al. (2020) demonstrated no difference in fasting blood sugar levels across the avocado, low‐fat, high‐fat, and moderate‐fat diets.

Over time, normal values for high blood pressure have changed, and food may be a significant factor (Cheng et al. 2017; Evbayekha et al. 2022). We studied the effect of avocado intake on blood pressure and found that it significantly decreased SBP while increasing DBP; however, this was not statistically significant.

According to Pieterse et al. (2005) there was no weight loss or avocado (MUFA) effect on arterial compliance, plasma fibrinogen, serum lipid levels, SBP, or DBP. However, any possible effects on these variables might have been obscured by the small sample size. Mahmassani et al. (2018) previously did a meta‐analysis that looked at the relationship between avocado consumption and CVDs. According to the results, eating avocados may increase HDL cholesterol levels. However, avocado consumption had no significant effect on blood TC, LDL cholesterol, or TG levels.

The reason why some studies have reported a significant effect on some cardiac risk factors and some have not can be attributed to factors such as different duration and amount of avocado consumption in the studies, different clinical conditions in the studies, study design, age and gender of the participants, and baseline levels of the measured variables.

### 4.1 Study Limitations

The number of studies and the significant heterogeneity are two of the primary drawbacks of our meta‐analysis. However, by using the leave‐one‐out strategy, we were able to resolve the heterogeneity. Due to variances in variables such as participant types, dietary habits, control groups, and intervention duration, the analysis revealed notable discrepancies between trials. In accordance with the control group, we conducted a subgroup analysis, yielding more consistent outcomes. Another drawback is the limited sample sizes and notable fluctuation of results in the studies that were part of the study. This finding should, therefore, be interpreted cautiously, and more long‐term studies are required to completely comprehend how eating avocados affects blood lipid levels and other CVD risk factors. On the other hand, interpretation needs to be done carefully, given the high level of heterogeneity among studies on TG, TC, SBP, and DBP. The last limitation is that the protocol of the present study was not registered.

## 5 Conclusion

According to this meta‐analysis, avocado supplements raised DBP, TG, and TC while decreasing BMI, CRP, FBG, LDL, and SBP levels. To clarify the impact of avocado on CVD risk variables, particularly insulin resistance and inflammatory indicators, more carefully planned RCTs and mechanistic research are required. It appeared that taking avocado supplements for more than 6 weeks at a dose of about 300 mg/day was the most effective way to reduce DBP. Furthermore, a drop in DBP levels was observed following 6 weeks of avocado consumption, indicating a non‐linear relationship between the length of the avocado intervention and DBP. Finally, it is recommended that studies with a stronger design, with longer‐term interventions and different doses, and with larger populations with different clinical conditions be conducted to obtain more robust results regarding avocado consumption on human health.

## Author Contributions

**Sahel Hamednia:** writing – original draft (equal). **Zahra Shouhani:** writing – original draft (equal). **Sara Tavakol:** writing – original draft (equal). **Nazanin Montazeri:** writing – original draft (equal). **Mohammadreza Amirkhan‐Dehkordi:** writing – original draft (equal). **Mohammad Amin Karimi:** writing – original draft (equal). **Mehraveh Bastamkhani:** writing – original draft (equal). **Haleh Chavoshian Tabrizy:** writing – review and editing (equal). **Ramin Amirsasan:** writing – original draft (equal). **Javad Vakili:** writing – original draft (equal). **Mehdi Karimi:** writing – review and editing (equal). **Omid Asbaghi:** writing – original draft (equal).

## Disclosure

Registration: The protocol of this systematic review and meta‐analysis was not registered.

## Ethics Statement

The authors have nothing to report.

## Consent

The authors have nothing to report.

## Conflicts of Interest

The authors declare no conflicts of interest.

## Supporting information


Table S1.

## Data Availability

The datasets generated during and/or analyzed during the current study are available from the corresponding author upon reasonable request.

## References

[fsn370547-bib-0001] Asbaghi, O. , F. Fouladvand , M. J. Gonzalez , D. Ashtary‐Larky , R. Choghakhori , and A. Abbasnezhad . 2021. “Effect of Green Tea on Glycemic Control in Patients With Type 2 Diabetes Mellitus: A Systematic Review and Meta‐Analysis.” Diabetes and Metabolic Syndrome: Clinical Research and Reviews 15, no. 1: 23–31. 10.1016/j.dsx.2020.11.004 33285391

[fsn370547-bib-0002] Asbaghi, O. , F. Naeini , D. Ashtary‐Larky , et al. 2021. “Effects of Chromium Supplementation on Blood Pressure, Body Mass Index, Liver Function Enzymes and Malondialdehyde in Patients With Type 2 Diabetes: A Systematic Review and Dose‐Response Meta‐Analysis of Randomized Controlled Trials.” Complementary Therapies in Medicine 60: 102755. 34237387 10.1016/j.ctim.2021.102755

[fsn370547-bib-0003] Begg, C. B. , and J. A. Berlin . 1988. “Publication Bias: A Problem in Interpreting Medical Data.” Journal of the Royal Statistical Society. Series A, Statistics in Society 151, no. 3: 419–445.

[fsn370547-bib-0004] Borenstein, M. , L. V. Hedges , J. P. Higgins , and H. R. Rothstein . 2010. “A Basic Introduction to Fixed‐Effect and Random‐Effects Models for Meta‐Analysis.” Research Synthesis Methods 1, no. 2: 97–111. 26061376 10.1002/jrsm.12

[fsn370547-bib-0005] Borenstein, M. , L. V. Hedges , J. P. Higgins , and H. R. Rothstein . 2021. Introduction to Meta‐Analysis. John Wiley & Sons.

[fsn370547-bib-0006] Cheng, H. M. , G. Koutsidis , J. K. Lodge , A. Ashor , M. Siervo , and J. Lara . 2017. “Tomato and Lycopene Supplementation and Cardiovascular Risk Factors: A Systematic Review and Meta‐Analysis.” Atherosclerosis 257: 100–108. 28129549 10.1016/j.atherosclerosis.2017.01.009

[fsn370547-bib-0007] DerSimonian, R. , and N. Laird . 1986. “Meta‐Analysis in Clinical Trials.” Controlled Clinical Trials 7, no. 3: 177–188. 3802833 10.1016/0197-2456(86)90046-2

[fsn370547-bib-0008] Dreher, M. L. , and A. J. Davenport . 2013. “Hass Avocado Composition and Potential Health Effects.” Critical Reviews in Food Science and Nutrition 53, no. 7: 738–750. 23638933 10.1080/10408398.2011.556759 PMC3664913

[fsn370547-bib-0009] Egger, M. , G. D. Smith , M. Schneider , and C. Minder . 1997. “Bias in Meta‐Analysis Detected by a Simple, Graphical Test.” BMJ 315, no. 7109: 629–634. 9310563 10.1136/bmj.315.7109.629 PMC2127453

[fsn370547-bib-0010] Evbayekha, E. O. , O. E. Okobi , T. Okobi , et al. 2022. “The Evolution of Hypertension Guidelines Over the Last 20+ Years: A Comprehensive Review.” Cureus 14, no. 11: e31437. 36523741 10.7759/cureus.31437 PMC9746526

[fsn370547-bib-0011] Hasanloei, M. A. V. , M. Rahimlou , A. Eivazloo , S. Sane , P. Ayremlou , and R. Hashemi . 2020. “Effect of Oral Versus Intramuscular Vitamin D Replacement on Oxidative Stress and Outcomes in Traumatic Mechanical Ventilated Patients Admitted to Intensive Care Unit.” Nutrition in Clinical Practice 35, no. 3: 548–558. 10.1002/ncp.10404. 31486158

[fsn370547-bib-0012] Hashemi, R. , A. Mehdizadeh Khalifani , M. Rahimlou , and M. Manafi . 2020. “Comparison of the Effect of Dietary Approaches to Stop Hypertension Diet and American Diabetes Association Nutrition Guidelines on Lipid Profiles in Patients With Type 2 Diabetes: A Comparative Clinical Trial.” Nutrition and Dietetics 77, no. 2: 204–211. 10.1111/1747-0080.12543. 31162810

[fsn370547-bib-0013] Hernández Salazar, M. , A. Flores , E. Ramírez , J. Llaca Díaz , B. Rodríguez , and H. Castro . 2022. “Effect of Avocado Honey on Anthropometric and Biochemical Parameters in Healthy Subjects: A Pilot Randomised Controlled Trial.” CyTA Journal of Food 20, no. 1: 78–85.

[fsn370547-bib-0014] Kopin, L. , and C. Lowenstein . 2017. “In the Clinic Dyslipidemia.” Annals of Internal Medicine 167, no. 11: ITC81–ITC96. 29204622 10.7326/AITC201712050

[fsn370547-bib-0015] Lichtenstein, A. H. , P. M. Kris‐Etherton , K. S. Petersen , et al. 2022. “Effect of Incorporating 1 Avocado Per Day Versus Habitual Diet on Visceral Adiposity: A Randomized Trial.” Journal of the American Heart Association 11, no. 14: e025657. 35861827 10.1161/JAHA.122.025657 PMC9707833

[fsn370547-bib-0016] Liu, X. , J. Sievert , M. L. Arpaia , and M. A. Madore . 2002. “Postulated Physiological Roles of the Seven‐Carbon Sugars, Mannoheptulose, and Perseitol in Avocado.” Journal of the American Society for Horticultural Science 127, no. 1: 108–114.

[fsn370547-bib-0039] Mahmassani, H. A. , E. E. Avendano , G. Raman , and E. J. Johnson . 2018. “Avocado Consumption and Risk Factors for Heart Disease: A Systematic Review and Meta‐analysis.” American Journal of Clinical Nutrition 107, no. 4: 523–536. 29635493 10.1093/ajcn/nqx078

[fsn370547-bib-0017] Mahmoud, A. M. , R. J. Hernandez Bautista , M. A. Sandhu , and O. E. Hussein . 2019. “Beneficial Effects of Citrus Flavonoids on Cardiovascular and Metabolic Health.” Oxidative Medicine and Cellular Longevity 2019, no. 1: 5484138. 30962863 10.1155/2019/5484138 PMC6431442

[fsn370547-bib-0018] Martínez‐Abundis, E. , M. González‐Ortiz , A. R. Mercado‐Sesma , C. Reynoso‐von‐Drateln , and A. Moreno‐Andrade . 2013. “Effect of Avocado Soybean Unsaponifiables on Insulin Secretion and Insulin Sensitivity in Patients With Obesity.” Obesity Facts 6, no. 5: 443–448. 24135894 10.1159/000355720 PMC5644760

[fsn370547-bib-0019] Mas‐Capdevila, A. , J. Teichenne , C. Domenech‐Coca , et al. 2020. “Effect of Hesperidin on Cardiovascular Disease Risk Factors: The Role of Intestinal Microbiota on Hesperidin Bioavailability.” Nutrients 12, no. 5: 1488. 32443766 10.3390/nu12051488 PMC7284956

[fsn370547-bib-0020] Matthan, N. R. , L. Lovato , K. S. Petersen , et al. 2024. “Effect of Daily Avocado Consumption for 6 Mo Compared With Habitual Diet on Red Blood Cell Fatty Acid Profiles and Association With Cardiometabolic Risk Factors in Individuals With Abdominal Obesity: A Randomized Trial.” American Journal of Clinical Nutrition 120, no. 4: 794–803. 39128497 10.1016/j.ajcnut.2024.08.002

[fsn370547-bib-0021] Mitchell, M. N. 2012. Interpreting and Visualizing Regression Models Using Stata. Vol. 558. Stata Press.

[fsn370547-bib-0022] Muralidhara, B. M. , T. Sakthivel , K. S. Shivashankara , et al. 2023. “Morpho‐Biochemical Characterization of a Unique Avocado (*Persia americana* Mill.) Accession PA‐026 (IC0644455).” Journal of Horticultural Sciences 18, no. 2: 1675.

[fsn370547-bib-0023] Naseri, K. , S. Saadati , D. Ashtary‐Larky , et al. 2022. “Probiotics and Synbiotics Supplementation Improve Glycemic Control Parameters in Subjects With Prediabetes and Type 2 Diabetes Mellitus: A GRADE‐Assessed Systematic Review, Meta‐Analysis, and Meta‐Regression of Randomized Clinical Trials.” Pharmacological Research 184: 106399. 35987483 10.1016/j.phrs.2022.106399

[fsn370547-bib-0024] Noormohammadi, M. , G. Eslamian , S. Malek , N. Shoaibinobarian , and S. N. Mirmohammadali . 2022. “The Association Between Fertility Diet Score and Polycystic Ovary Syndrome: A Case‐Control Study.” Health Care for Women International 43, no. 1–3: 70–84. 33797335 10.1080/07399332.2021.1886298

[fsn370547-bib-0025] Pacheco, L. S. , Y. Li , E. B. Rimm , et al. 2022. “Avocado Consumption and Risk of Cardiovascular Disease in US Adults.” Journal of the American Heart Association 11, no. 7: e024014. 35352568 10.1161/JAHA.121.024014 PMC9075418

[fsn370547-bib-0026] Page, M. J. , D. Moher , P. M. Bossuyt , et al. 2021. “PRISMA 2020 Explanation and Elaboration: Updated Guidance and Exemplars for Reporting Systematic Reviews.” BMJ (Clinical Research Edition) 372: n160. 10.1136/bmj.n160 PMC8005925 33781993

[fsn370547-bib-0027] Pieterse, Z. , J. Jerling , W. Oosthuizen , et al. 2005. “Substitution of High Monounsaturated Fatty Acid Avocado for Mixed Dietary Fats During an Energy‐Restricted Diet: Effects on Weight Loss, Serum Lipids, Fibrinogen, and Vascular Function.” Nutrition 21, no. 1: 67–75. 15661480 10.1016/j.nut.2004.09.010

[fsn370547-bib-0028] Ruiz‐Moreno, C. , B. Lara , J. J. Salinero , D. Brito de Souza , J. M. Ordovás , and J. Del Coso . 2020. “Time Course of Tolerance to Adverse Effects Associated With the Ingestion of a Moderate Dose of Caffeine.” European Journal of Nutrition 59: 3293–3302. 31900579 10.1007/s00394-019-02167-2

[fsn370547-bib-0029] Scott, T. M. , H. M. Rasmussen , O. Chen , and E. J. Johnson . 2017. “Avocado Consumption Increases Macular Pigment Density in Older Adults: A Randomized, Controlled Trial.” Nutrients 9, no. 9: 919. 28832514 10.3390/nu9090919 PMC5622679

[fsn370547-bib-0030] Souza Fernandes Azevedo, A. C. , M. F. Cardoso de Lima , E. L. Lopes Ramos , A. P. Boroni Moreira , and C. Teodoro de Souza . 2023. “Effects of Avocado Oil Supplementation on Lipid Profile and Atherogenic Indices in a Doubleblind and Randomised Intervention in Patients With Metabolic Syndrome.” Demetra: Alimentação, Nutrição & Saúde 18: 70457.

[fsn370547-bib-0031] Sterne, J. A. C. , J. Savović , M. J. Page , et al. 2019. “RoB 2: A Revised Tool for Assessing Risk of Bias in Randomised Trials.” BMJ (Clinical Research Edition) 366: l4898. 10.1136/bmj.l4898. 31462531

[fsn370547-bib-0032] US Department of Agriculture and US Department of Health and Human Services . 2010. Nutrition and Your Health: Dietary Guidelines for Americans. US Department of Agriculture and US Department of Health and Human Services.

[fsn370547-bib-0033] Vaduganathan, M. , G. A. Mensah , J. V. Turco , V. Fuster , and G. A. Roth . 2022. “The Global Burden of Cardiovascular Diseases and Risk: A Compass for Future Health.” Journal of the American College of Cardiology 80: 2361–2371. 36368511 10.1016/j.jacc.2022.11.005

[fsn370547-bib-0035] Wang, L. , L. Tao , L. Hao , et al. 2020. “A Moderate‐Fat Diet With One Avocado Per Day Increases Plasma Antioxidants and Decreases the Oxidation of Small, Dense LDL in Adults With Overweight and Obesity: A Randomized Controlled Trial.” Journal of Nutrition 150, no. 2: 276–284. 31616932 10.1093/jn/nxz231 PMC7373821

[fsn370547-bib-0034] Wang, L. , P. L. Bordi , J. A. Fleming , A. M. Hill , and P. M. Kris‐Etherton . 2015. “Effect of a Moderate Fat Diet With and Without Avocados on Lipoprotein Particle Number, Size and Subclasses in Overweight and Obese Adults: A Randomized, Controlled Trial.” Journal of the American Heart Association 4, no. 1: e001355. 25567051 10.1161/JAHA.114.001355 PMC4330060

[fsn370547-bib-0036] Wegier, A. , F. Lorea Hernández , A. Contreras , W. Tobón , and A. Mastretta‐Yanes . 2017. “*Persea americana*” (Errata Version Published in 2018). The IUCN Red List of Threatened Species.

[fsn370547-bib-0037] Zhang, X. , D. Xiao , G. Guzman , I. Edirisinghe , and B. Burton‐Freeman . 2022. “Avocado Consumption for 12 Weeks and Cardiometabolic Risk Factors: A Randomized Controlled Trial in Adults With Overweight or Obesity and Insulin Resistance.” Journal of Nutrition 152, no. 8: 1851–1861. 35700149 10.1093/jn/nxac126 PMC9486596

[fsn370547-bib-0038] Zhao, L. , D. K. Ingram , E. Gumpricht , et al. 2023. “Effects of an Unripe Avocado Extract on Glycaemic Control in Individuals With Obesity: A Double‐Blinded, Parallel, Randomised Clinical Trial.” Nutrients 15, no. 22: 4812. 38004206 10.3390/nu15224812 PMC10674186

