# Supplementary material for: Effects of Avocado Products on Cardiovascular Risk Factors in Adults: A GRADE‐Assessed Systematic Review and Meta‐Analysis
Source: Food Sci Nutr. 2025 Jul 2;13(7):e70547. doi: 10.1002/fsn3.70547 (PMC12221997; doi:10.1002/fsn3.70547)
Supplement: Supplementary file 1 — Table S1. [file FSN3-13-e70547-s001.docx]

Supplementary table 1. Search strategy from databases (up to May 2025)

| Terms | |  |
| --- | --- | --- |
| "Avocado" OR "Persea" OR "Persea americana" OR "Alligator pear" and "randomized" OR "random" OR "randomly" OR "placebo" OR "randomized controlled trial" OR "randomized clinical trial" OR "RCT "OR "blinded" OR "double blind" OR "double blinded" OR "Cross-Over" OR "parallel" | |  |
| Search strategy | |  |
| WEB OF SCIENCES | "Avocado" OR "Persea" OR "Persea americana" OR "Alligator pear" (Topic) AND "randomized" OR "random" OR "randomly" OR "placebo" OR "randomized controlled trial" OR "randomized clinical trial" OR "RCT "OR "blinded" OR "double blind" OR "double blinded" OR "Cross-Over" OR "parallel" (Topic) | 392 |
| PUBMED | ("Avocado"[Title/Abstract] OR "Persea"[Title/Abstract] OR "Persea americana"[Title/Abstract] OR "Alligator pear"[Title/Abstract]) AND ("randomized"[Title/Abstract] OR "random"[Title/Abstract] OR "randomly"[Title/Abstract] OR "placebo"[Title/Abstract] OR "randomized controlled trial"[Title/Abstract] OR "randomized clinical trial"[Title/Abstract] OR "RCT "OR "blinded"[Title/Abstract] OR "double blind"[Title/Abstract] OR "double blinded"[Title/Abstract] OR "Cross-Over"[Title/Abstract] OR "parallel"[Title/Abstract]) | 187 |
| Scopus | (TITLE-ABS-KEY ("Avocado" OR "Persea" OR "Persea americana" OR "Alligator pear") AND TITLE-ABS-KEY ("randomized" OR "random" OR "randomly" OR "placebo" OR "randomized controlled trial" OR "randomized clinical trial" OR "RCT " OR "blinded" OR "double blind" OR "double blinded" OR "Cross-Over" OR "parallel")) | 579 |
| All |  | 1158 |
